# Comparative Metagenomic Analysis of Bacteriophages and Prophages in Gnotobiotic Mouse Models

**DOI:** 10.3390/microorganisms12020255

**Published:** 2024-01-25

**Authors:** Oluwaseun A. Ishola, Susanne Kublik, Abilash Chakravarthy Durai Raj, Caspar Ohnmacht, Stefanie Schulz, Bärbel U. Foesel, Michael Schloter

**Affiliations:** 1 Research Unit for Comparative Microbiome Analysis, Helmholtz Zentrum München—German Research Center for Environmental Health, 85764 Neuherberg, Germany; oluwaseunisholaayodeji@gmail.com (O.A.I.);; 2 Mucosal Immunology Group, Center of Allery and Environment (ZAUM), Technical University of Munich, Helmholtz Zentrum München, 85764 München, Germany; 3 Chair for Environmental Microbiology, TUM School of Life Science, Technical University of Munich, 85354 Freising, Germany; 4 Central Institute for Nutrition and Health, Technical University of Munich, 85354 Freising, Germany

**Keywords:** murine models, virome, prophages, bacteriophages, metagenomics, auxiliary metabolic genes (AMGs)

## Abstract

Gnotobiotic murine models are important to understand microbiota–host interactions. Despite the role of bacteriophages as drivers for microbiome structure and function, there is no information about the structure and function of the gut virome in gnotobiotic models and the link between bacterial and bacteriophage/prophage diversity. We studied the virome of gnotobiotic murine Oligo-MM12 (12 bacterial species) and reduced Altered Schaedler Flora (ASF, three bacterial species). As reference, the virome of Specific Pathogen-Free (SPF) mice was investigated. A metagenomic approach was used to assess prophages and bacteriophages in the guts of 6-week-old female mice. We identified a positive correlation between bacteria diversity, and bacteriophages and prophages. *Caudoviricetes* (82.4%) were the most prominent class of phages in all samples with differing relative abundance. However, the host specificity of bacteriophages belonging to class *Caudoviricetes* differed depending on model bacterial diversity. We further studied the role of bacteriophages in horizontal gene transfer and microbial adaptation to the host’s environment. Analysis of mobile genetic elements showed the contribution of bacteriophages to the adaptation of bacterial amino acid metabolism. Overall, our results implicate virome “dark matter” and interactions with the host system as factors for microbial community structure and function which determine host health. Taking the importance of the virome in the microbiome diversity and horizontal gene transfer, reductions in the virome might be an important factor driving losses of microbial biodiversity and the subsequent dysbiosis of the gut microbiome.

## 1. Introduction

The growing interest to study the role and dynamics of the human gut microbiome in health has triggered the development of suitable animal models [1,2]. Murine models are widely accepted due to their high similarities to the human genome (about 90% of genes and their functions are the same in humans), resulting in a comparable physiology and anatomical structure, particularly of the gastrointestinal tract [1]. Furthermore, subjects in murine models are simple to maintain, and fast to breed, and genetic modifications can be introduced easily. Finally, the microbiomes of the murine and human gut are comparable (at least on the phyla level) and are dominated by *Firmicutes* and *Bacteriodetes* [2,3].

Therefore, the development of gnotobiotic mouse models based on defined microbial consortia can provide insight into how combinations of thoroughly characterized microbes influence the host and subsequently health and disease [2]. One of the most used microbial consortia for gnotobiotic mice is the Altered Schaedler flora (ASF) developed for intestinal studies in the 1970s [3]. This consortium is stable and of low diversity, comprising eight anaerobic bacterial species that represent the most abundant phyla of the murine gut microbiota [3]. Though they have been criticized for their low complexity, gnotobiotic murine models inoculated with ASF have been successfully used to demonstrate that microbial antigens are necessary for the homeostatic development of mucosal T cells in the colonic lamina propria [4]. Recently, it became possible to establish several additional microbial consortia in the murine gut in a stable manner. Among those consortia, the Oligo-mouse microbiota consortium (Oligo-MM12), made up of a defined cultivable bacterial community of 12 species (representing the five most prevalent murine intestine bacterial phyla), has become of high interest as it offers long-term colonization stability across multiple generations [5,6,7]. In addition, Oligo-MM12 mounts significant resistance against the colonization of pathogenic *Salmonella typhimurium* [7], potentially out-competing invasive species for the ecological niche and/or due to interactions of the introduced microbial consortia with host cells. 

Although the use of artificial microbial consortia in gnotobiotic murine models has been well established in human microbiome research, there is limited knowledge regarding the diversity of viruses, particularly bacteriophages in these models. These viruses may significantly influence the effects of the introduced microbiota, impacting their abundance, phenotype, and genome structure [8]. The virome is primarily composed of bacteriophages, prophages, rarer eukaryotic viruses, and endogenous retroviruses [9]. Studies have reported that the virome community shape the dynamics and diversity of the bacterial population through lytic interaction, horizontal gene transfer, encoded virulence proteins, and metabolic genes [8,10,11,12,13]. 

In this study, we investigated the virome of two well-established gnotobiotic murine models, namely Oligo-MM12 (12 bacterial species) and reduced Altered Schaedler Flora (ASF, three bacterial species) and compared the results to the virome of specific pathogen-free (SPF) wild-type mice. We further analyzed the potential impact of different virome structures on the dynamics of horizontal gene transfer amongst bacteria via transduction. We hypothesize that prophages (integrated viruses), and bacteriophages (viruses capable of infecting bacteria) are present in the gnotobiotic mouse models, and their diversity is associated with the complexity of the introduced microbial communities. Virome analysis was conducted using whole-genome sequencing followed by bioinformatics-based reconstruction of the various virome components. 

## 2. Materials and Methods

### 2.1. Mouse Models and Sampling

All procedures involving animals were performed according to local ethical and regulatory guidelines, which complied with the EU regulations regarding research on experimental animals. The animal study was reviewed and approved by the Regierung von Oberbayern, Germany (Regierung von Oberbayern; approval number 55.2-1-54-2532-192-2016). All mice models studied in the frame of this publication were inoculated with their respective bacterial inoculum in the lab of our collaborators (see Acknowledgement). 

We used C57BL/6J-derived female mice harboring the Oligo-MM12 bacterial community (Table S1) with twelve bacterial species [7], and the reduced Altered Schaedler Flora (ASF), with three bacterial species (Table S1), respectively [7,14]. For comparison, C57BL/6J female wild-type SPF mice were used. The gnotobiotic and SPF mice were born with their respective microbiota. During rearing, the different mice of each model were kept separately in germ–free isolators to prevent cross-contamination. Wood chips were used as bedding and mice were fed a sterile regular chow diet and delivered ddH_2_O orally with a 0.22 µm sterile syringe [5]. Four mice per group were used for each microbial condition. After six weeks, the animals were sacrificed by cervical dislocation, and the tissue and lumen contents of the ileum and colon of each mouse were obtained resulting in 48 samples (3 murine models × 2 gut compartments × 2 fractions per gut compartment × 4 replicates), which were snap-frozen at −80 °C for further analysis. To further increase DNA concentrations for metagenomic sequencing, replicates were pooled, resulting in 12 samples. 

### 2.2. DNA Extraction

Total genomic DNA was extracted from each sample using a phenol–chloroform-based co-extraction protocol with slight modifications [15]. In brief, 0.1 g material was homogenized using a precelleys24 homogenizer (Bertin Technologies, Montigny-le-Bretonneux, France) in 120 mM sodium phosphate (NaPO_4_) pH 8.0 and TNS pH 8.0 buffer; 500 mM Tris-HCL pH 8.0, 100 mM sodium chloride and 10% sodium dodecyl sulfate (*w*/*v*) buffer for 30 s at 5.5 m/s and centrifuged for 10 min at 16,000× *g*. The supernatant was transferred into an RNase-free tube and nucleic acids were stepwise purified. First, an equal amount of phenol/chloroform/isoamyl alcohol solution (25:24:1, pH 8.0) was added. Second, chloroform/isoamyl alcohol (24:1 (vol/vol)) was added. Each step was followed by vortexing, centrifugation (5 min at 16,000× *g*) and transferring the upper phase to fresh tubes. DNA was precipitated by adding 2 volumes of a 10% (*w*/*v*) polyethylene glycol and 1.2 M NaCl solution and incubated for 2 h on ice. After centrifugation (16,000× *g*, 10 min, 4 °C), the pellet was washed in 500 µL ice-cold DNase/RNase free 70% EtOH, centrifuged again (16,000× *g*, 10 min, 4 °C), air-dried, and eluted in 50 µL of DEPC-treated (0.1% *v*/*v*) milliQ water. The eluted co-extracted nucleic acids were quantified on a Qubit 4 Fluorometer using the Qubit dsDNA/RNA BR Assay Kit (ThermoFisher Scientific, Waltham, MA, USA) according to the manufacturer’s instructions. The purity was assessed by measuring the A260 nm/A280 nm and A260 nm/A230 nm ratios using a NanoDrop 1000 spectrophotometer (ThermoFisher Scientific, MA, USA). Two (2 µg) of the nucleic acid extracts were digested using the RNase I kit (Invitrogen, Waltham, CA, USA) to degrade the RNA. The pure DNA was then quantified with the Qubit BR assay kit and stored at −20 °C until further processing.

### 2.3. Metagenomic Library Preparation and Sequencing

DNA concentrations of all samples were adjusted to a final amount of 10 ng of DNA for metagenomic library preparation with the NEBNext Ultra II FS DNA Library Prep Kit and NEBNext Multiplex Oligos for Illumina (New England Biolabs, Ipswich, MA, USA). Library preparation was performed according to the manufacturer’s instructions with a few modifications. In brief, the DNA was fragmented using enzyme digestion (New England Biolabs, MA, USA) for 15 min and the Illumina adapters were diluted 25-fold to 0.6 µM to avoid the formation of primer dimers. Size selection was conducted with Agencourt AMPure XP beads (Beckman Coulter, Brea, CA, USA), to select fragments of 250–400 bp length, purify libraries and eliminate residual primer-dimers (1:0.6 DNA to bead ratio). The indexing PCR was performed with 10 cycles. Library size and concentration were evaluated using a 5200 Fragment analyzer system (Agilent, Santa Clara, CA, USA) and a DNF-473 Standard Sensitivity NGS Fragment Analysis Kit (Agilent, CA, USA). Libraries were pooled equimolarly (final concentration of 4 nM), and 17 pM of the mixture was spiked with 1% PhiX and sequenced on NextSeq Illumina platform (Illumina Inc., San Diego, CA, USA) using the paired-end mode 2 × 150 bp Kit v 2.5 (Illumina Inc., CA, USA).

### 2.4. Bioinformatic Analysis Workflow 

#### 2.4.1. Quality Filtering and Bacterial Taxonomic Assignment

**Figure 1:**
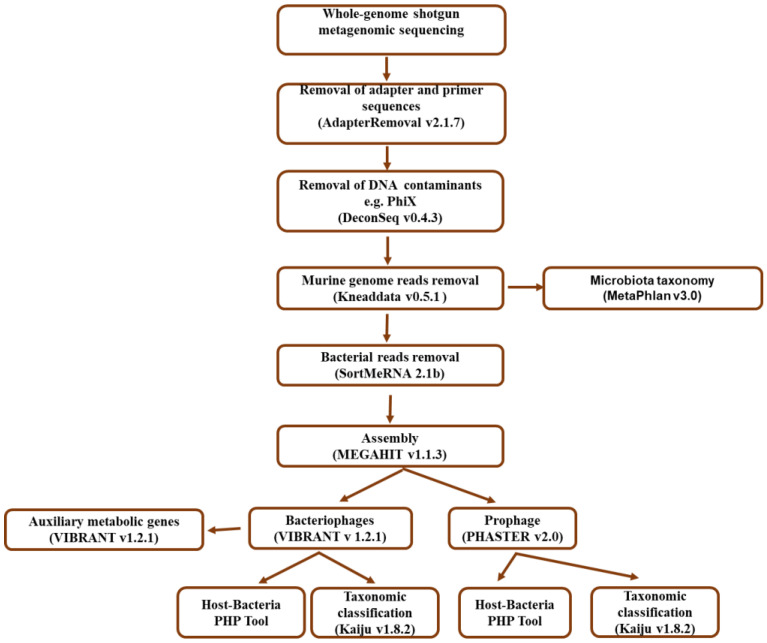
Bioinformatic workflow used for prophage and bacteriophage characterization. Raw reads from the metagenome dataset were processed by filtering the reads based on read quality and length. The reads were de novo assembled to produce contigs that were subjected to ORF predictions, transfer RNA (tRNA)insertion site identification and a clustering algorithm of phage-like genes to detect prophage. The bacteriophage mechanism of identification is based on protein annotation signatures using neural networks from non-reference-based similarity searches with Hidden Markov Models (HMMs), as well as a unique “v-score” metric for diverse and novel virus mining. Taxonomic composition, functional roles and putative bacterial hosts were also evaluated.

The bioinformatic analysis workflow is shown in Figure 1. The number of reads from demultiplexed raw data was counted with FastQC version v0.11 (https://www.bioinformatics.babraham.ac.uk/projects/fastqc/ accessed on 7 June 2022). For raw data quality control, the guidelines from [16] were followed. Adapter sequences were removed with AdapterRemoval v2.1.7, accessed on 8 June 2022 [17]; low-quality bases with Phred quality scores lower than 30 were trimmed and reads shorter than 50 bp were discarded at the same step. PhiX contamination was removed with DeconSeq v0.4.3, accessed on 8 June 2022 [18]. Reads identified as derived from the murine genome were removed from the datasets by mapping against NCBI C57BL/6J mouse database reference genome (National Centre for Biotechnology Information) using Kneaddata v0.5.1 (http://huttenhower.sph.harvard.edu/kneaddata accessed on 14 July 2022). Next, bacterial taxonomy was assigned by profiling the remaining non-mouse-reads with MetaPhlan v3.0 on galaxy web (https://usegalaxy.eu/ accessed on 6 June 2023), which is based on unique clade-specific marker genes for bacteria [19,20]. This step was performed to analyze bacterial diversity in the respective samples of SPF, Oligo-MM12 and ASF. 

The reads obtained for tissue and content for each gut compartment and murine model were further concatenated, due to the low number of non–mouse reads in the tissue samples. In the next step, paired end reads were assembled for each sample with MEGAHIT v1.1.3, with a contig minimum length set to 200 bp [21]. After assembling, non-assembled reads were mapped back to the contigs of each colon and ileum library to calculate coverage. 

#### 2.4.2. Bacteriophage Identification and Taxonomic Classification 

Bacteriophage-designated contigs were identified and annotated using VIBRANT v1.2.1 (Virus Identification by IteRative ANnoTation) [22]. The minimum contig length was set to 1000 bp and a minimum of four open reading frames was mandatory. VIBRANT uses the Hidden Markov Model (HMM) to screen against the protein families (Pfam), Kyoto Encyclopedia of Genes and Genomes (KEGG), and Virus Orthologous Groups (VOG) databases [22] to identify bacteriophages. VIBRANT also uses CheckV and virus orthologous groups (VOGs) metrics of nucleotide replication and viral hallmark protein calculation to estimate bacteriophage contig quality. All identified bacteriophage contigs from the samples were clustered and dereplicated using CD-HIT-EST v4.8.1, accessed on 19 April 2023 [23] at 99% identity to remove duplicated sequences and to allow for the identification of bacteriophage-specific contigs. Using a homology-based matching approach, predicted bacteriophage contigs were submitted to Kaiju v1.8.2 [24] using the NCBI RefSeq viral-only database (version 2023.05.26) in greedy mode, with an e-value threshold of ≤10^−3^ for taxonomic classification. Relative abundance was calculated using contig coverage. Putative bacterial hosts of the identified bacteriophage contigs were predicted with prokaryotic virus–host predictor (PHP) (http://www.computationalbiology.cn/phageHostPredictor/home.html, accessed on 21 April 2023) [25] based on sequence homology and the JGI IMG Viral Database of cultivated and uncultivated viruses’ version 3.0 (https://img.jgi.doe.gov/cgi-bin/vr/main.cgi) (>2,000,000 viral genome fragments, accessed on 11 July 2021) using default settings (identity ≥ 30%, E-value ≤ 10^−5^). The taxonomic classification was conducted at the family/class level according to the recently updated viral nomenclature [26].

To identify viral auxiliary metabolic genes (AMGs), VIBRANT v1.2.1 was run on CD-HIT-EST bacteriophage contigs. Outputs were binned into metabolic pathways by searching the Pfam accession number of each gene in the KEGG database [22]. The potential interaction pattern between the predicated bacterial hosts and bacteriophages was calculated with relative abundance values using random network by Cytoscape. 

#### 2.4.3. Prediction and Taxonomic Classification of Prophages

To predict the taxonomy of the prophages in our murine models, we only used contigs >2000 bp in length according to [27]. Prophages were recovered using the Phage Search Tool Enhanced Release (PHASTER v2.0) web server (https://phaster.ca/, accessed 18 April 2023). Contigs were assigned using a custom database that combines protein and phage sequences from the National Centre for Biotechnology Information (NCBI) and prophage sequences using BLAST [28,29]. The identified prophages were classified as complete (100%) and incomplete (<90%) according to the DBSCAN clustering algorithm used by PHASTER [30]. The completeness score calculation was based on the proportion of phage genes on the prophage designated contig, which is associated with capsid, head, tail, coat, portal, and phage regulation genes, and integrase, transposase, terminase, and functional genes such as lysin and bacteriocin. Using a homology-based matching approach, predicted prophage contigs were submitted to Kaiju using the NCBI RefSeq viral-only database (version 2023.05.26) [24] in greedy mode, with an e-value threshold of ≤10^−3^ [31] for taxonomic classification. Relative abundance was calculated using prophage-designated contig coverage. In detail, coverage was calculated by mapping the metagenomic quality-controlled reads to prophage-assigned contigs (after filtering bacterial genes and reads associated with the murine genome). The number of mapped reads was divided by the total number of contigs length to obtain the fraction of reads that represent prophages (prophage designated mapped reads/total contig length). Putative bacterial hosts were predicted by submitting the outputted prophage-designated contigs to prokaryotic virus–host predictor (PHP) (http://www.computationalbiology.cn/phageHostPredictor/home.html, accessed on 1 June 2023), which is a tool based on a gaussian model that relies on differences of k-mer frequencies and homology between virus and bacteria genomic sequences. The output is a score which is the logarithm of the probability of being a viral host. The highest-consensus host is then identified with the NCBI taxonomy ID of profiled phage [25]. In addition, we assessed the original hosts of the prophages in Oligo-MM12 and ASF. Therefore, we blasted individual contigs against available genome sequences for bacterial strains of Oligo-MM12 and ASF using BlastN [32]. The similarity percentage was set at >95% [33].

The host–bacteria ratio of prophage-designated contigs in the murine models were predicted by aligning identified prophage contigs from the murine model gut compartments to the gnotobiotic microbiota individual genome prophages. The percentage of similarity was calculated [34]. The potential interaction pattern between the predicted bacterial hosts and prophages was calculated with the relative abundance values using random network by Cytoscape. 

### 2.5. Statistical Analysis

Statistical analyses were performed in R [35]. The Shannon diversity index [34] was calculated to estimate alpha diversity using the Vegan v2.6-4 [36] package. The Shannon diversity of the bacterial community at the family-level taxonomic classification was calculated on the species level by using the command line library (vegan), viralshannon <- diversity (prophages, index = “shannon”). For prophages and bacteriophages, the family-level taxonomic classification was used to ensure uniform comparison of the gnotobiotic mice with SPF. Visualization was performed using the statistical software R (https://www.r-project.org/, accessed from 2 January 2022) packages ggplot2, reshape2, and plyr for the stacked bar plots and GraphPad Prism v5.0 for scatter plots (Graph Pad Software, La Jolla, CA, USA). The tables were designed with Microsoft Office. Host–bacteria and phage interaction/relatedness were calculated and visualized with Cytoscape v3.10.0 (https://cytoscape.org/download.html, accessed on 8 August 2023) using a random layout network of node algorithm showing a predictive interaction between the bacteriophage/prophages and the predicted host bacteria [37]. 

## 3. Results

### 3.1. Sequencing Statistics

The total number of raw reads obtained from the 12 metagenomic libraries was 304,255,379 million reads and ranged between 10,159,586 and 33,212,147 million paired end reads per sample, respectively (Table S2). As sequencing was performed without murine host depletion, the percentage of murine genome-contaminating reads was high (>70% in most samples; Table S2), and they were excluded during sample processing. Merging of the gut compartment lumen and tissue reads after quality control resulted in 83,212,848 million paired-end reads in total.

The metagenomic assembly resulted in 413,974 contigs (size ranging from 200 bp to 420,970 bp) ranging between 5457 and 304,899 contigs per sample (Table S3). Contig length and number varied as expected based on sequencing depth and bacterial diversity. However, all murine models followed a similar contig size distribution (Figure S1) and reached saturation when plotting the contig index (number of contigs from longest to the shortest) against cumulative contigs length that showed the size of an assembly and the number of contigs in it (Figure S2). 

### 3.2. Bacterial Diversity in the Two Gut Compartments of the Three Murine Models

While the bacterial community composition of Oligo-MM12 and ASF is well defined, the microbiota of the SPF model used in our study was unknown and most likely triggered by several environmental factors like animal housing, diet and age of the animals and gender. Phylogenetic profiling was carried out on non-mouse-reads before assembly to demonstrate that the inoculated bacterial consortia were still present in the ASF and Oligo-MM12 gnotobiotic mice and to assess the diversity of bacteria in the used SPF mice. On the family level, as expected bacterial diversity was higher in SPF compared to ASF and Oligo-MM12.

In ASF, three species were used as inoculum, namely *Lactobacillus murinus*, *Parabacteriodetes goldsteinii* and *Clostridium* sp. These were all detected in the respective gut samples but differed in their abundance in response to the investigated gut compartment. *Lactobacillus murinus* was dominant in the ileum while *Parabacteroides goldstenii* prevailed in higher relative abundance in the colon (Table S4). 

Ten of the twelve inoculated species were found in the Oligo-MM12 model, where their distribution varied between the gut compartments. The most abundant species in the colon were *Akkermansia muciniphila*, followed by *Muribaculum intestinale* and *Turicimonas muris.* In contrast, *Bacteriodes caecimuris* dominated the ileum. *Acutalibacter muris* and *Bifidobacterium longum* subsp. *animalis YL2* were undetected in the ileum and colon, although they were part of the inoculum used (Table S4). No overlap between ASF and Oligo-MM-12 based murine models were found.

In SPF, two hundred and fifty-eight bacterial species belonging to thirty-one bacterial families, respectively were profiled, and their distribution also varied between gut compartments (Table S5). *Muribaculaceae* was the most abundant, with higher dominance in the ileum compared to the colon, followed by *Bacteriodetes* in the ileum and *Firmicutes* in the colon (Table S6). Bacterial species that overlapped between SPF and Oligo-MM12 included *Turicimonas muris*, *Clostridium clostridioforme Blautia coccoide*, and *Bacteriodes caecimuris*, while the species that overlapped between SPF and ASF was *Parabacteroides goldsteinii.*

### 3.3. Bacteriophages and Predicted Bacterial Hosts

A total of 986 bacteriophage contigs were assembled from metagenomic reads obtained from the three murine models. In ASF, 0.93% of the total reads were assigned to bacteriophages; this number was slightly increased for Oligo-MM12 and SPF, with 2.84%, and 4.24% of the reads, respectively (Table S7). For all models, more contigs linked to bacteriophages were detected in the colon compared to the ileum (Figure S3). Based on genome completeness, 0.03% of the contigs were rated as high quality, 1.72% as medium quality, and 97.9% as low quality (Figure S3). 

**Figure 2:**
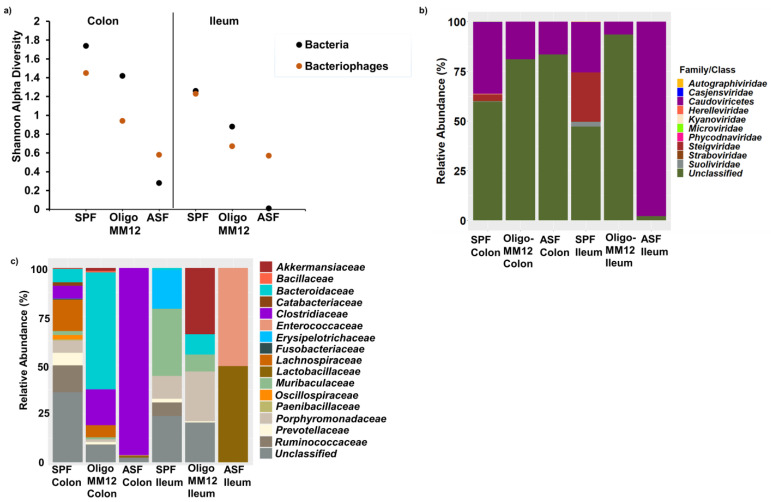

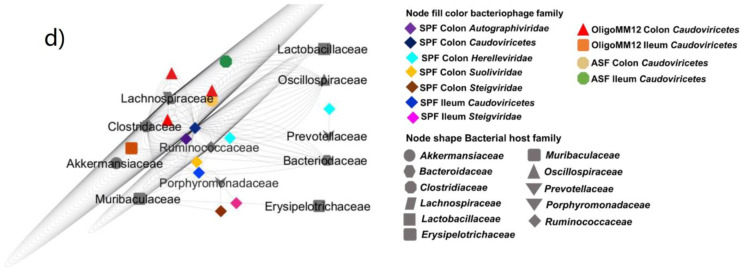
Taxonomy of bacteriophages and putative bacterial host families in ileum and colon samples from the gnotobiotic murine models inoculated with Oligo-MM12 and ASF as well as from SPF mice, (**a**) Shannon alpha diversity of bacteriophages and bacteria assessed at the family level. (**b**) Community composition of bacteriophages. (**c**) Relative abundance of the predicted host-bacterial families, (**d**) Networks between bacteria and bacteriophages depicting their interactions, displaying bacterial phylogeny, bacteriophage families and murine models. The link line (edges) denotes oligonucleotide homology similarity frequency used to predict the respective bacterial hosts.

In our study, we observed that the diversity of bacteriophages increased with increasing bacterial diversity (Figure 2a). Diversity was generally higher in colon samples especially in SPF. The bacteriophage contigs were taxonomically assigned to nine families and one class (Figure 2b), for some contigs, viral hallmark genes were detected, but lineages could not be further classified. Bacteriophages belonging to class *Caudoviricetes* dominated all samples with different relative abundances. In ASF, class *Caudoviricetes* showed a higher relative abundance in the ileum samples compared to the colon; *unclassified* were more abundant. Unknown families were only detected in colon samples with a high frequency. In both Oligo-MM12 gut compartments, class *Caudoviricetes* dominated more in the colon compared to the ileum. Contigs that could not be assigned to the family level were present in high abundance in the colon and ileum samples. In SPF, the classes *Caudoviricetes* and *Steigviridae* were equally abundant in the ileum. In addition, some contigs which could not be further assigned were found. The highest diversity was found in the colon of SPF mice. Here, *Casjensviridae*, *Herelleviridae*, *Autographiviridae*, *Kyanoviridae*, *Straboviridae*, *Suoliviridae*, *Phycodnaviridae* and *Microviridae* were detected, but in relatively lower abundances compared to class *Caudoviricetes*. Also, in these samples from SPF mice, some contigs could not be further assigned to the family and class level. 

Overall, the diversity of potential host bacteria for the identified bacteriophages was higher in the colon than in the ileum, and bacteriophages from SPF displayed broader host range characteristics compared to the gnotobiotic models mainly in the colon (Figure 2c,d). Figure 2d illustrates the putative host–bacteriophage interaction pattern in the model’s gut compartments for easier visualization.

Interestingly, the predicted hosts for the class *Caudoviricetes* differed in the three murine models under investigation, attributing the differences in the bacteriophages on the genus, species, or strain level. In ASF, *Lactobacillaceae* were identified as potential hosts for the class *Caudoviricetes* in the ileum. In contrast, in colon samples of ASF mice, the identified hosts for the class *Caudoviricetes* were mostly *Clostridiaceae*. In Oligo-MM12, the class *Caudoviricetes* was assigned to *Clostridiaceae* and *Lachnospiraceae* in the colon; in the ileum, potential hosts for the class *Caudoviricetes* were *Bacteroidaceae* and *Akkermansiaceae.* In SPF, bacteriophage families were associated with *Lachnospiraceae* and *Ruminococcaceae*, mainly in the colon, whereas in the ileum *Muribaculaceae* was identified as the potential host. *Autographiviridae* phages in the SPF colon were assigned mostly to *Ruminococcaceae*.

### 3.4. Taxonomy of Prophages and Their Predicted Bacteria Host 

From the three murine models, a total of 43 prophage-designated contigs were identified, ranging from 2.8 to 53 kb in size (Table S8) with varying completeness (Figure S4). In the ASF, 0.80% of all reads were assigned to prophages, and comparable numbers were found for Oligo-MM12 and SPF (0.45% and 0.63%, respectively; Table S7). The highest number of prophage-designated contigs of 29 were detected in the SPF model, particularly in the colon region, while 10 were found in ASF and 4 in Oligo-MM12. 

From the known sequence of Oligo-MM12 and ASF microbiota, a total of 50 and 19 prophage contigs with varying completeness (Table S9) were identified, respectively. 

**Figure 3:**
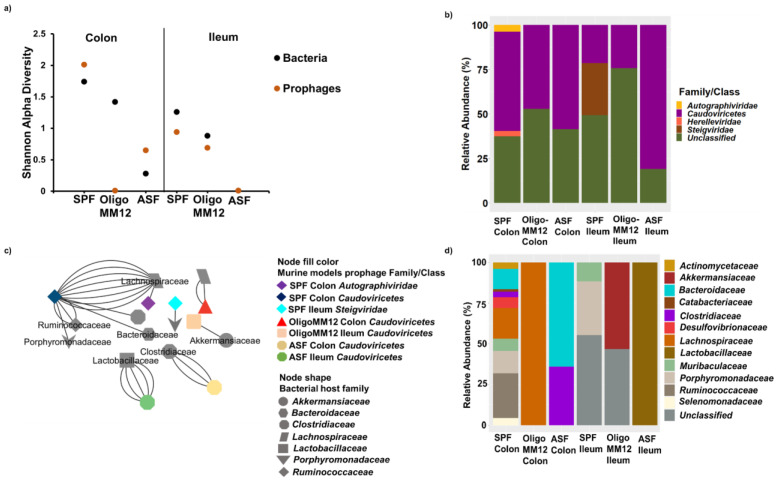
Taxonomy of prophages and putative bacterial host families in ileum and colon samples from the gnotobiotic murine models inoculated with Oligo-MM12 and ASF as well as from SPF mice. (**a**) Shannon alpha diversity of prophages and predicted bacterial hosts at the family level; (**b**) taxonomic composition of prophages; and (**c**) bacteria–prophage interaction network analysis displaying the phylogeny of the bacterial host phage family linked to the investigated murine models and the two gut compartments. The link line (edges) denotes the oligonucleotide homology similarity frequency used to predict the host bacteria. (**d**) Relative abundance of the predicted host-bacteria of prophage contigs.

As for bacteriophages, prophage diversity increased with the increasing complexity of the gut microbiome, except for the Oligo-MM12 colon (Figure 3a). 

Prophage contigs were taxonomically classified into 3 families and 1 class. Similar to bacteriophages, some contigs were identified as of prophage origin but could not be further assigned to the family level. Again, the class *Caudoviricetes* was the predominating class of prophages in all three murine models (Figure 3b). 

In ASF, the class *Caudoviricetes* showed a higher relative abundance in the colon samples compared to the ileum. The unknown family’s relative abundance increased in colon samples. In Oligo-MM12, the class *Caudoviricetes* was specific to both gut compartments. In SPF, the class *Caudoviricetes* was more abundant in the colon than in the ileum samples. In addition, *Steigviridae* was specific to the ileum, whereas *Herelleviridae* and *Autographiviridae* were specific to the colon samples. Some contigs which could not be further assigned to the family level were present in both the colon and ileum. 

In ASF, predicted bacterial hosts for class *Caudoviricetes* were again *Lactobacillaceae* classified *Lactobacillus murinus* in the ileum, and *Clostridiaceae* classified *Clostridium* sp. in the colon. In Oligo-MM12, bacterial hosts for class *Caudoviricetes* were *Lachnospiraceae* classified *Clostridium innocuum* and *Clostridium clostridioforme* in the colon, and *Akkermansiaceae* classified *Akkermansia muciniphila* in the ileum. Like for bacteriophages, host bacteria diversity was higher in SPF, especially in the colon. The class *Caudoviricetes* interacted mainly with *Lachnospiraceae* and *Ruminococcaceae* in the colon, whereas *Porphyromonadaceae* were identified as bacterial hosts for *Steigviridae* in the ileum (Figure 3c,d). 

To identify potential host bacteria for the identified phages, bacterial genomes of OMM12 and ASF were blasted against the generated contigs for the identified phages. Most of the generated contigs showed similarity levels with the genome of host bacteria from the two models used of between 80 and 92.20%, which is too varied for a robust predication of potential host bacteria; two contigs could not at all been aligned, indicating that during cultivation of the bacterial in the different labs for inoculation, new phage infections took place. Only four prophages that were detected in the Oligo-MM12 colon and ASF ileum shared 100% similarity with the respective inoculated bacteria, namely *Clostridium innocuum* in Oligo-MM12 and *Lactobacillus murinus* in ASF (Table S10).

### 3.5. Auxiliary Metabolic Genes (AMGs) of Bacteriophages

**Figure 4:**
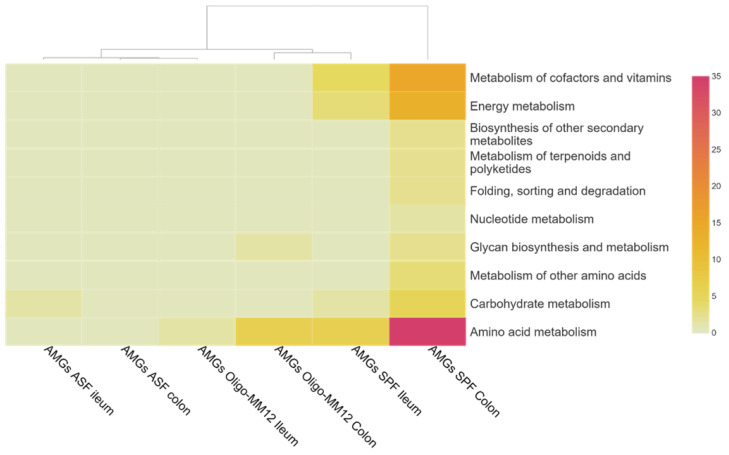
Bacteriophage-associated auxiliary metabolic genes functional categories of KEGG pathways in ileum and colon samples from the gnotobiotic murine models inoculated with Oligo-MM12 and ASF as well as from SPF mice. Darker colors represent higher level of count while lighter shade signifies lower count.

The identified auxiliary metabolic genes encoded on the bacteriophage-designated contigs were grouped into ten KEGG metabolic categories (Figure 4). The DNA cytosine methyltransferase gene (*dcm*) involved in cysteine and methionine metabolism was found in all models except ASF, with the highest count in SPF colon (Figure S5). The *dcm* gene was prevalent on *Caudoviricetes* in SPF, and most likely derived from infections with *Ruminococcaceae* and *Lachnospiraceae*, which correlated with the putative bacterial hosts predicted in both SPF gut compartments (Figure 2c). In Oligo-MM12, the *dcm* gene found in both the colon and ileum was linked to *Bacteroidaceae*, one of the probable bacterial hosts.

The genes *nadM* and *NAMPT*, which encode for nicotinamide nucleotide adenylyl transferase and nicotinamide phosphoribosyl transferase, respectively, are known to drive the metabolism of cofactors and vitamins through folate biosynthesis, and nicotinate and nicotinamide metabolism were found on contigs assigned to *Caudoviricetes* in the SPF colon and ileum. These genes were likely acquired through their interaction with *Ruminococcaceae* in the SPF colon; however, the uptake route in the ileum is not clear. 

The only AMG found in ASF was *iolU*, (scyllo-inositol 2-dehydrogenase), which is involved in carbohydrate metabolism and specific to *Caudoviricetes* (Figure 4 and Figure S5). The AMG *cysH* (phosphoadenosine phosphosulfate reductase) involved in sulfur metabolism was specific to SPF and connected to *Caudoviricetes* in the colon and ileum, acquired through potential interaction from *Ruminococcaceae* in the colon and *Muribaculaceae* in the ileum. Overall, a higher count of AMGs was found in SPF, particularly in the colon over in the gnotobiotic models, and the abundance of AMGs was linked to the increasing complexity of the gut microbiome.

## 4. Discussion

Bacteriophages are the most prevalent part of the virome in almost all ecosystems, including mammal guts [38,39]. Their interactions with the microbiota shape the composition, abundance and functioning of the microbial community. For example, as a result of antagonistic interactions and by lowering the abundance of specific bacteria through lysis, strain-specific phages can reduce Oligo-MM12 microbiota’s capacity to resist *Salmonella typhimurium* infection [40]. There are few studies involving humans, but in soil and aquatic environments, mutual phage–host interactions can enhance host bacterial metabolic processes and thereby improve environmental fitness, which stimulate phage replication, particularly in stressful conditions [41,42]. However, the association between bacterial and viral (prophage and bacteriophage) communities in the gut is not fully understood. Using gnotobiotic murine models Oligo-MM12 and ASF, we could prove that increased bacterial diversity is linked to increased bacteriophage/prophage diversity and higher auxiliary gene transfer between bacteriophages and their hosts. 

### 4.1. Gnotobiotic Oligo-MM12 and ASF Microbiota Consortia Were Detected with Differing Relative Abundances in the Gut

Oligo-MM12 and ASF bacterial consortia were detected in the gut after 6 weeks of rearing, except for *Acutalibacter muris* and *Bifidobacterium longum* subsp. *animalis* in Oligo-MM12. These results were consistent with a previous study where the stable and long-term colonization of inoculated consortia in Oligo-MM12 and ASF were reported [5]. The undetectability of *Acutalibacter muris* could be because it has been considered as a late colonizer of the gut and consequently may be present only in low numbers in our sample, derived from relatively young animals 6 weeks of age. In contrast, the abundance of *Bifidobacterium longum* subsp. *animalis YL2* was reported to rapidly decline in Oligo-MM12 one week after inoculation, as this bacterium was described as a very early colonizer and mainly found in the neonatal gut, where it contributes to the reduction in anaerobic organisms [43,44]. In addition, we could prove that bacterial relative abundance varied in the colon and ileum of all murine models [45].

### 4.2. Bacterial Diversity Triggers Bacteriophage and Prophage Diversity 

The application of state-of-the-art bioinformatics tools led to the identification of unique prophage- and bacteriophage-designated contigs in the gut of the gnotobiotic models Oligo-MM12 and ASF, and wildtype SPF mice. Prophages and bacteriophages also differed significantly across models and gut compartments, indicating a high degree of specificity for compartment and model. This is in agreement with studies that have shown that the virome community is specialized and dependent on many factors, where the genetics of the host as well as the microbiome are only one of many bacterial community similarities [12,46].

The majority of contigs assigned to the prophage and bacteriophage belonged to the viral class of head tailed *Caudoviricetes* (dsDNA), dominating in all models. This is in agreement with other studies from murine and human guts, as well as other ecosystems. dsDNA *Caudoviricetes* are ubiquitous and have a wide range of bacterial hosts [45,47,48,49,50]. Intestinal *Caudoviricetes*-classified bacteriophages can broaden their adaptation and host ranges through encoded diversity-generating retro-elements [51]. This supports our finding on bacteriophage’s broader bacterial hosts found in all models. Also, a study showed that specific *Caudoviricetes*-classified phages can be a biocontrol agent against *Salmonella enterica* serovar enteritidis [52]. The high proportion of unclassified viruses in samples from the SPF mice suggests a large knowledge gap in the taxonomy of intestinal viruses, thereby highlighting the need for more viral metagenomic studies.

The observed bacteriophage diversity increased with increasing gut microbiota bacterial diversity (SPF > Oligo-MM12 > ASF), suggesting that microbiota richness is a strong determinant of bacteriophage diversity. This trend has also been described for monozygotic twins and healthy adults [12,53]. Given the dynamics of microbiota–phage interactions, the precise mechanism differs in all ecosystems because these interactions are driven by variable factors such as environmental conditions, microbiome density, host genetics and nutrition [54,55,56]. Anyhow, when bacterial diversity is low, fewer species of bacteria are accessible for phages to infect; thus, expanding microbiota richness is beneficial for bacteriophages because it provides a broader host pool for infection and replication to synthesize more virions. This observation is consistent with findings from other environments. In a study in soils, with increasing soil depth bacterial richness decreased, affecting phage abundance and diversity [57]. In another study using soils, increases in the soil virome were triggered by the bacterial abundance and diversity but not by growth and activity [57,58]. Furthermore, our findings support the ‘piggyback the winner’ concept. This concept states that lysogenic association between putative bacterial hosts and temperate phages predominate in a dense, rich and healthy gut environment. Low bacterial diversity causes low phage diversity, favoring a lysogenic lifestyle [59,60]. In most lysogenic associations, the phage integrates and multiplies vertically with the host, and gets incorporated into the next generation [60,61,62].

Similarly, prophage diversity correlated with microbiota richness in all models. Prophages are temperate phages that integrate into their hosts in a stable manner. Since lysogeny is the primary mode of phage contact with commensal bacteria in the gut [61], their increasing diversity with microbiota complexity suggests either ongoing lysogenic infection in the murine models or could be related to an infection with prophages already before the bacteria has reached the gut. Also, most of the profiled prophages are temperate. The number of lysin genes, which code for enzymes that catalyze the degradation of the cell wall of the host bacterium, facilitating the release of phage particles, was significantly reduced. In addition, the recovery of mostly fragmented prophages could suggest a loss of sequence, which may affect genes necessary for lytic infection [33,62]. Prophages showed prominent interaction with the *Lachnospiraceae* and *Ruminococcaceae* families in our study. The long-term coexistence of prophages and microbiota can induce genomic variations and strain heterogeneity in murine models over time, which could compromise the reproducibility of murine model investigations. For example, the differences between *Salmonella enterica* serovars typhi and typhimurium were attributed to genetic variations induced by prophages [63]. The predicted putative bacterial hosts of the identified prophages and bacteriophages reflect their bacterial microbiota community in the gut of Oligo-MM12 and ASF, indicating the expected close interaction of prophages with their bacterial host. Even though the bacterial hosts of the prophages were similar to the bacteria microbiota, the similarity alignment revealed disparity between Oligo-MM12 and ASF genome and profiled murine prophages. This could be attributed to low sequencing coverage and probable genetic variability. Genetic variability causes the degradation, shorten, fragmentation and alteration of integrated viruses. This is induced by mutations, genetic rearrangements, massive DNA deletions and environmental selection pressure [33,64]. A study reported the gradual loss of lambda prophage sequences in *E. coli* after environmental selection [64].

### 4.3. AMGs Are Associated with Microbiota Complexity

Phages can influence microbiota fitness and adaptation to new environmental conditions through the horizontal gene transfer of auxiliary metabolic genes (AMGs) [65]. In our study, we could also find AMG, which were encoded on contigs identified as being part of the phage. We found that the diversity of AMGs associated with bacteriophages was again linked to the microbial diversity of the gut microbiota (SPF > Oligo-MM12 > ASF). Since higher microbiota diversity provides a broader pool of potential hosts for phages as well as genes which can be horizontally transferred, this result is in line with our expectations. 

The identified AMGs were encoded by the dominant bacteriophage-classified *Caudoviricetes*, correlating with other studies [65,66]. The most common AMG present in samples derived from Oligo-MM12 and SPF mice was *dcm*, which is a gene encoding for an enzyme catalyzing cysteine and methionine degradation where it methylates the internal C in sequence 5′CCWGG3. The *dcm* gene has been identified to be highly conserved in environment such as marine sediments [22,67]. The increased abundance of *dcm* has been reported in human GIT, where it mediates the degradation of organosulfur compounds such as methionine into sulfide during infection or cell lysis [68]. This process enhances the growth of specific organisms, increases nutrient availability for hosts and provides fitness benefits to the phages. The synthesized sulfide can also be used for amnio acid synthesis to provide energy source [69]. The *cysH* gene encodes for an enzyme involved in assimilatory sulfate reduction pathway that incorporates sulfides into cysteine [70], and its presence in SPF suggests viral involvement in sulfur cycling, which has implications on the health of the gastrointestinal tract. It may alter biogeochemical processes in other environments like water and soil [60]. In addition, studies have shown that *cysH* may serve as a fitness modulator during sulfur-limiting periods in *Burkholderia*-phage infection and help pig gut viruses adapt to the complex gut environment and homeostasis [71,72,73]. Nutrient availability, including other factors, influences the distribution of *dcm* and *cysH* in different ecosystems [68]. The AMGs linked to amino acid metabolism in SPF and Oligo-MM12 are capable of mediating the synthesis of SCFAs from peptides, which serves as an energy source to improve microbiome stability [74,75].

## 5. Conclusions

Our study gives basic insights into the gastrointestinal virome of important gnotobiotic murine models, host-bacterial composition and associated AMGs. Our data indicate that a higher diversity of bacteria microbiota triggers an increased diversity of bacteriophages and prophages. Taking the importance of the virome as a stabilizing factor for the microbiome into account (“killing the winner”) as well as the importance for horizontal gene transfer and adaptation, it is obvious that reductions in the virome might be an important factor driving losses of microbial biodiversity and the subsequent dysbiosis of the gut microbiome. In our study, we analyzed microbial models with 3 and 12 bacterial strains only and compared them to those of wild-type SPF mice, which is important for model description but may not mimic shifts in the gut microbiome, e.g., as a result of diet or other environmental triggers. Here, future research is needed, also implementing data from large-scale cohorts.

## Data Availability

Illumina sequence data providing the basis for this article are available in the Sequence Read Archive (SRA) repository under the BioSample accession numbers as part of the BioProject ID PRJNA943611, SRA accession: SUB12941023 at http://www.ncbi.nlm.nih.gov/sra/ accessed on 14 December 2023.

## Supplementary Materials

The following supporting information can be downloaded at: https://www.mdpi.com/article/10.3390/microorganisms12020255/s1.
