# Supplementary material for: Comparative Metagenomic Analysis of Bacteriophages and Prophages in Gnotobiotic Mouse Models
Source: Microorganisms. 2024 Jan 25;12(2):255. doi: 10.3390/microorganisms12020255 (PMC10892684; doi:10.3390/microorganisms12020255)
Supplement: Supplementary file 1 [file microorganisms-12-00255-s001.zip › microorganisms-2800384-supplementary.pdf]

Comparative Metagenomic Analysis of Bacteriophages and Prophages in Gnotobiotic Mouse Models

Oluwaseun A. Ishola,[1] Susanne Kublik,[1] Abilash Chakravarthy Durai Raj,[1†] Caspar Ohnmacht,[2] Stefanie Schulz[1*], Bärbel U. Foesel[1††], Michael Schloter,[1,3,4]

[1] Research Unit for Comparative Microbiome Analysis, Helmholtz Center Munich, German Research Center for Environmental Health, Neuherberg, Germany.

[2] Mucosal Immunology Group, Center of Allery and Environment (ZAUM), Technical University and Helmholtz Center Munich, Munich, Germany.

[3] Technical University of Munich, Chair for Environmental Microbiology, Freising, Germany.

[4] Technical University of Munich, Central Institute for Nutrition and Health, Freising, Germany.

[†] Present address: Max von Pettenkofer Institute of Hygiene and Medical Microbiology, Ludwig-Maximilians-University of Munich, Munich, Germany.

[††] Present address: Institute of Epidemiology, Research Unit Molecular Epidemiology, Helmholtz Munich, Germany.

Table S1: Gnotobiotic murine models Oligo-MM12 and ASF microflora inoculum

**Oligo-MM12**	**ASF**
Clostridium innocuum	*Clostridium spp ASF*
Bacteroides caecimuris	*Lactobacillus murinus ASF*
Lactobacillus reuteri	*Parabacteriodetes goldsteinii*
Enterococcus faecalis	
Muribaculum intestinale	
Flavonifractor plautii	
Clostridium clostridioforme	
Akkermansia muciniphila	
Turicimonas muris	
Blautia coccoides	
Acutalibacter muris	
Bifidobacterium animalis subsp. animalis	

Table S2: Read statistics of metagenomic sequencing samples included in this study.

**Sample Sequencing name**	**Sample name**	**No. of raw reads**	**No. of reads after adapter removal**	**No. of reads after trimming**	**No. of host-mouse reads**	**No. of non-mouse reads**	**% of murine reads**	**No. of 16S rRNA removed**	**No. of non-rRNA reads**
seuntube10_S10_R1_001, pair,clean,fq	SPF ileum content R1	32469279.00	28187328.00	26789345.00	19849360.00	6701229.00	74.09	57003.50	6644225.50
seuntube10_S10_R2_001, pair,clean,fq	SPF ileum content R2	32469279.00	28187328.00	26789345.00	19849360.00	6701229.00	74.09	57003.50	6644225.50
**seuntube11_S11_R1_001,pair1,clean,fq**	ASF ileum content R1	22518571.00	19158564.00	14528890.00	13775112.00	597319.00	94.81	20252.00	577067.00
**seuntube11_S11_R2_001,pair1,clean,fq**	ASF ileum content R2	22518571.00	19158564.00	14528890.00	13775112.00	597319.00	94.81	20252.00	577067.00
**seuntube12_S12_R1_001,pair1_clean,fq**	Oligo-MM ileum content R1	26300108.00	24650174.00	24650174.00	21868625.00	2293007.00	88.72	112982.50	2180024.50
**seuntube12_S12_R2_001,pair1_clean,fq**	Oligo-MM ileum	26300108.00	24650174.00	24650174.00	21868625.00	2293007.00	88.72	112982.50	2180024.50

	content R2								
**seuntube1_S1_R1_001,pair1_clean,fq**	SPF colon content R1	33212147.00	29625432.00	22775691.00	1050952.00	21701930.00	4.61	137837.00	21564093.00
**seuntube1_S1_R2_001,pair1_clean,fq**	SPF colon content R2	33212147.00	29625432.00	22775691.00	1050952.00	21701930.00	4.61	137837.00	21564093.00
**seuntube2_S2_R1_001,pair1_clean,fq**	ASF colon content R1	24930096.00	23388936.00	22386511.00	3425486.00	18898931.00	15.30	98294.50	18800636.50
**seuntube2_S2_R2_001,pair1_clean,fq**	ASF colon content R2	24930096.00	23388936.00	22386511.00	3425486.00	18898931.00	15.30	98294.50	18800636.50
**seuntube3_S3_R1_001,pair1_clean,fq**	Oligo-MM colon content R1	23217211.00	22227299.00	20412986.00	1214256.00	19142525.00	5.95	106352.00	19036173.00
**seuntube3_S3_R2_001,pair1_clean,fq**	Oligo-MM colon content R2	23217211.00	22227299.00	20412986.00	1214256.00	19142525.00	5.95	106352.00	19036173.00
**seuntube4_S4_R1_001,pair1_clean,fq**	SPF ileum tissue R1	20201473.00	16104339.00	15024334.00	13479765.00	2346752.00	89.72	114821.50	2231930.50

**seuntube4_S4_R2_001,pair1_clean,fq**	SPF ileum tissue R2	20201473.00	16104339.00	15024334.00	13479765.00	2346752.00	89.72	114821.50	2231930.50
**seuntube5_S5_R1_001,pair1_clean,fq**	ASF ileum tissue R1	27893268.00	21613834.00	20208117.00	18010960.00	1996069.00	89.13	33724.50	1962344.50
**seuntube5_S5_R2_001,pair1_clean,fq**	ASF ileum tissue R2	27893268.00	21613834.00	20208117.00	18010960.00	1996069.00	89.13	33724.50	1962344.50
**seuntube6_S6_R1_001,pair1_clean,fq**	Oligo-MM ileum tissue R1	31028385.00	26791872.00	21849264.00	17294225.00	4134271.00	79.15	214795.50	3919475.50
**seuntube6_S6_R2_001,pair1_clean,fq**	Oligo-MM ileum tissue R2	31028385.00	26791872.00	21849264.00	17294225.00	4134271.00	79.15	214795.50	3919475.50
**seuntube7_S7_R1_001,pair1_clean**	SPF colon tissue R1	21879263.00	20682534.00	19758590.00	16036821.00	2368853.00	81.16	52517.50	2316335.50
**seuntube7_S7_R2_001,pair1_clean**	SPF colon tissue R2	21879263.00	20682534.00	19758590.00	16036821.00	2368853.00	81.16	52517.50	2316335.50
**seuntube8_S8_R1_001,pair1_clean**	ASF colon tissue R1	30445992.00	27888628.00	27888628.00	22815114.00	3678338.00	81.81	183416.50	3494921.50

**seuntube8_S8_R2_001,pair1_clean**	ASF colon tissue R2	30445992.00	27888628.00	27888628.00	22815114.00	3678338.00	81.81	183416.50	3494921.50
**seuntube9_S9_R1_001,pair1_clean**	Oligo-MM colon tissue R1	10159586.00	8007039.00	6097944.00	5530529.00	497985.00	90.69	12363.50	485621.50
**seuntube9_S9_R2_001,pair1_clean**	Oligo-MM colon tissue R2	10159586.00	8007039.00	6097944.00	5530529.00	497985.00	90.69	12363.50	485621.50
**Total**		**304255379.00**	**268325979.00**	**242370474.00**	**154351205.00**	**84357209.00**		**1144360.50**	**83212848.50**

Table S3: Contig assembly statistics of non-host non-rRNA reads.

Sample names	Total number of contigs	Total contigs length (bp)	Minimum contigs length	Maximum contigs length	Average length (bp)	N50
SPF merged colon content and tissue	304899	279317015	200	420970	916	1250
Oligo-MM12 merged colon content and tissue	12326	27776895	200	290252	2254	32819
ASF merged colon content and tissue	13979	15346287	200	355576	1098	34658
SPF merged ileum content and tissue	51777	59060320	200	147546	1141	2336
Oligo-MM12 merged ileum content and tissue	25536	19102394	200	237544	748	1442
ASF merged ileum content and tissue	5457	4140752	200	104512	759	5099

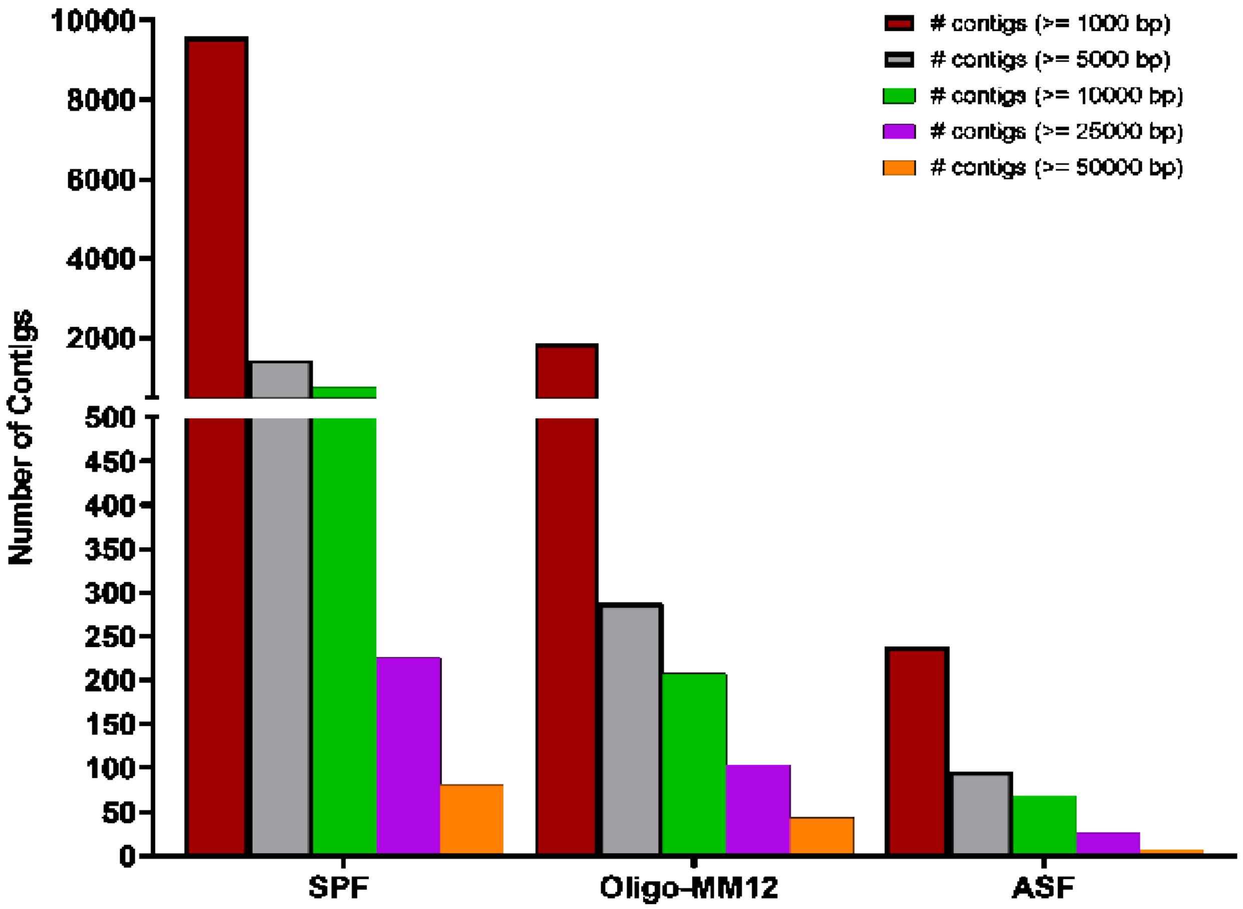

Figure S1: Assembly of murine model reads: Bar chart of paired-end contig assembly distribution in SPF, Oligo-MM12 and ASF.

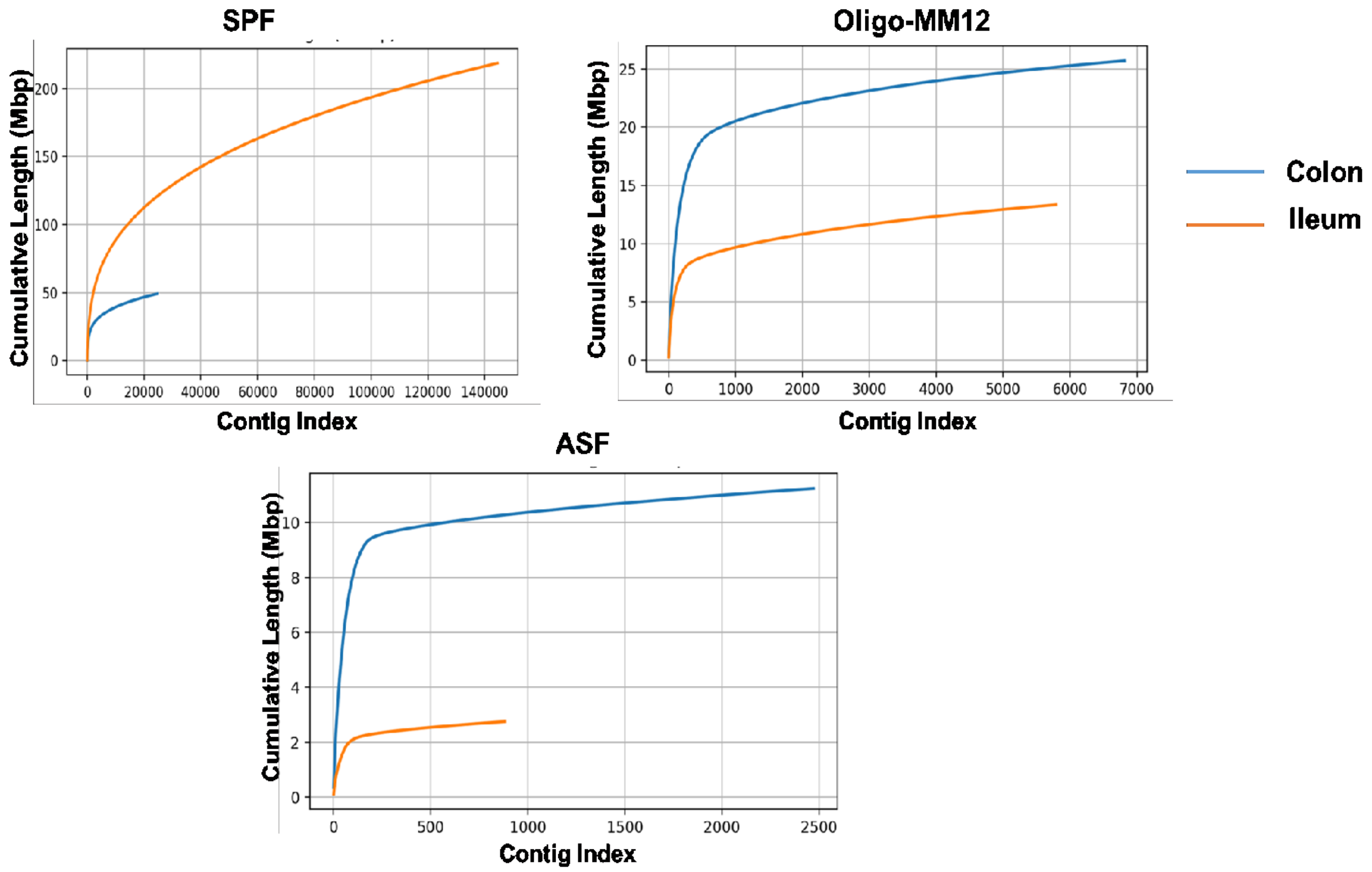

Figure S2: Assembly of murine model reads; frequency of assembled contigs distribution among the murine models. The cumulative length shows the size of an assembly and the number of contigs in it, and the contig index represents the longest to shortest assembly. Orange colour line represents the ileum while blue the colon.

Table S4: Bacterial community structure in ileum and colon samples from the gnotobiotic murine models inoculated with Oligo-MM12 and ASF. Values displayed are relative abundance in % at the species level.

Oligo-MM12			ASF		
**Bacterial species**	**Ileum**	**Colon**	**Bacterial species**	**Ileum**	**Colon**
Clostridium innocuum	0.13	0.07	*Clostridium spp ASF*	0	6.89
Bacteroides caecimuris	76.19	7.49	*Lactobacillus murinus ASF*	99.02	0.62
Lactobacillus reuteri	0.01	2.90	*Parabacteriodetes goldsteinii*	0.98	92.49
Enterococcus faecalis	0.01	0.00			
Muribaculum intestinale	10.62	30.66			
Flavonifractor plautii	0.49	0.32			
Clostridium clostridioforme	6.36	0.97			
Akkermansia muciniphila	3.27	39.67			
Turicimonas muris	2.76	17.62			
Blautia coccoides	0.16	0.30			
**Total**	**100**	**100**		**100**	**100**

Table S5: Bacterial community structure in ileum and colon samples from SPF mice. Values displayed are relative abundance in % at the species level.

**SPF bacterial species**	**Ileum**	**Colon**
Acutalibacter_muris	0	0.00455
Acutalibacter sp1XD833	0	0.0619
Akkermansia muciniphila	0.15498	0.12654
Alistipes sp	0.02773	3.45665
Anaerotruncus sp1XD4293	0	0.50146
Atopobiaceae bacteriumFL090493	0.0247	0.00568
Bacteria unclassifiedSGB41508	0	0.06453
Bacteria unclassifiedSGB41729	0	0.0288
Bacteria unclassifiedSGB43539	0	0.00414
Bacteria unclassifiedSGB43541	0	0.00386
Bacterium 01xD882	0	0.0103
Bacterium 1XD2113	0	0.03885
Bacterium 1XD421	0	0.52163
Bacterium 1XD4276	0	0.02923
Bacterium 1xD4287	0	0.22607
Bacterium 1xD848	0	0.02486
Bacterium 1xD86	0	0.14364
Bacterium D1629	0	0.05154
Bacterium D1676	0	0.02471
Bacteroidaceae bacterium	0.04505	1.72578
Bacteroidales bacterium	14.99742	4.74278
Bacteroides acidifaciens	0	0.14199
Bacteroides caecimuris	0	0.10552
Bacteroides spL104	0	0.43813
Barnesiella spCU968	3.28986	0.80602
BlautiaSGB43555	0	0.14114
Brevibacterium SGB41697	0	0.02819
Clostridiaceae bacterium	0	0.03219
Clostridiales bacterium	0	0.03974
Clostridium SGB40327	0.68585	0.25611
Desulfovibrio SGB40192	0.31103	10.83932
Desulfovibrio SGB41239	0	0.00324
Dorea sp52	0	0.01006
Duncaniella dubosii	7.6005	2.16354
Duncaniella freteri	1.99252	0.67867
Duncaniella muris	14.7991	4.48316

Emergencia sp1XD2110	0	0.00969
Eubacterium SGB102306 2	0	0.0142
GGB14001 SGB21428	1.0683	0.35265
GGB18827 SGB41440	0	0.0724
GGB20145 SGB41586	0	0.00422
GGB20146 SGB29427	0	0.0496
GGB20149 SGB29430	0	0.06222
GGB23844 SGB35575	0	1.10852
GGB24102 SGB40205	0	0.8953
GGB24132 SGB35935	1.23027	0.43477
GGB24149 SGB35953	2.87501	0.43156
GGB25052 SGB36974	0	0.67076
GGB25107 SGB41514	0	0.21614
GGB261 SGB59900	0	0.02212
GGB27782 SGB40196	0	0.5314
GGB27861 SGB40295	1.5944	0.19307
GGB27866 SGB40300	0.89879	0.43255
GGB27872 SGB40306	0.51612	0.18014
GGB27873 SGB40307	1.46125	0.51149
GGB27885 SGB40319	1.08366	0.38084
GGB27890 SGB40324	1.13688	0.45307
GGB27892 SGB40326	12.21319	4.11916
GGB27929 SGB40368	2.60108	0.878
GGB27929 SGB41624	0	0.00876
GGB28248 SGB40791	0.0543	2.22569
GGB28265 SGB40817	0	1.75288
GGB28308 SGB40878	0.04765	2.00762
GGB28317 SGB40889	0	0.17172
GGB28330 SGB40904	0	0.01785
GGB28357 SGB40935	0	0.15814
GGB28381 SGB40961	0	0.30219
GGB28382 SGB40962	0	0.42572
GGB28383 SGB40963	0.03231	0.18838
GGB28384 SGB40964	0	0.1056
GGB28386 SGB40966	0	0.13723
GGB28392 SGB40972	0	0.13631
GGB28393 SGB40973	0.03231	2.11221
GGB28394 SGB40975	0	0.01379
GGB28401 SGB40983	0	0.60913
GGB28403 SGB40190	0	0.14234
GGB28404 SGB40986	0	0.04308

GGB28409 SGB40991	0	0.01287
GGB28418 SGB41000	0	0.01072
GGB28418 SGB41001	0	0.68208
GGB28430 SGB41013	0	0.00254
GGB28431 SGB41014	0	0.00181
GGB28433 SGB41016	0	0.02539
GGB28437 SGB41020	0	0.02566
GGB28439 SGB41022	0	0.1858
GGB28456 SGB41039	0	0.24674
GGB28632 SGB41253	0	0.01488
GGB28761 SGB41409	0	0.0166
GGB28765 SGB41415	0	0.10759
GGB28769 SGB41421	0	0.00923
GGB28776 SGB41429	0	0.06031
GGB28782 SGB41435	0	0.02816
GGB28783 SGB41436	0	0.00492
GGB28793 SGB41446	0	0.02288
GGB28817 SGB41472	0	0.0245
GGB28822 SGB41478	0	0.00609
GGB28828 SGB41484	0	0.42117
GGB28829 SGB41485	0	0.07246
GGB28835 SGB41494	0	0.33478
GGB28836 SGB41495	0	0.00047
GGB28839 SGB41503	0	0.00146
GGB28841 SGB41505	0	0.01409
GGB28842 SGB41507	0	0.08936
GGB28845 SGB41512	0	0.02214
GGB28849 SGB41516	0	0.10228
GGB28853 SGB41519	0	0.02142
GGB28854 SGB41523	0	0.02539
GGB28855 SGB41524	0	0.03099
GGB28861 SGB41531	0	0.30702
GGB28865 SGB41537	0	0.0118
GGB28865 SGB43001	0	0.00704
GGB28868 SGB41542	0	0.17239
GGB28879 SGB43526	0	0.02334
GGB28880 SGB41560	0	0.06226
GGB28881 SGB41561	0	0.00972
GGB28883 SGB41564	0	0.03677
GGB28885 SGB41566	0	0.53526
GGB28888 SGB41569	0	0.0265

GGB28895 SGB41576	0	0.05296
GGB28901 SGB41592	0	0.06842
GGB28903 SGB41594	0	0.00927
GGB28904 SGB41597	0	0.0624
GGB28905 SGB41598	0	0.0457
GGB28907 SGB41600	0	0.11962
GGB28916 SGB41612	0	0.06896
GGB28927 SGB41625	0	0.0114
GGB28934 SGB41635	0	0.0236
GGB28949 SGB41655	0	0.06175
GGB28958 SGB41667	0	0.00766
GGB28959 SGB41668	0	0.02187
GGB28961 SGB41670	0	0.00247
GGB28967 SGB41417	0	0.0068
GGB28967 SGB41678	0	0.01306
GGB28980 SGB41692	0	0.07409
GGB28981 SGB41693	0	0.02798
GGB28981 SGB41694	0	0.00041
GGB28991 SGB41705	0	0.01749
GGB28996 SGB41712	0	0.50854
GGB29001 SGB41717	0	0.14935
GGB29002 SGB41718	0	0.19764
GGB29003 SGB41719	0	0.10879
GGB29004 SGB41721	0	0.56836
GGB29005 SGB41723	0	1.3658
GGB29008 SGB41728	0	1.04995
GGB29468 SGB42247	0.24007	0.01044
GGB29530 SGB42316	0	0.00513
GGB29531 SGB42317	0	0.22355
GGB29549 SGB42343	0	0.03529
GGB30141 SGB43066	0	0.02761
GGB30142 SGB43067	0	0.05018
GGB30145 SGB43072	0	0.05791
GGB30147 SGB43074	0	0.29976
GGB30290 SGB43252	0	0.46782
GGB30296 SGB43260	0	0.00408
GGB30298 SGB43262	0	0.00201
GGB30300 SGB43264	0	0.02247
GGB30301 SGB43265	0	0.00209
GGB30302 SGB43266	0	0.0349
GGB30302 SGB43267	0	0.03564

GGB30311 SGB43288	0	0.68854
GGB30408 SGB43438	0	0.00432
GGB30413 SGB43452	0	0.06032
GGB30414 SGB43453	0	0.01651
GGB30450 SGB43507	0	0.53368
GGB30451 SGB43508	0	0.05525
GGB30454 SGB43514	0	0.05656
GGB30455 SGB43519	0	0.1692
GGB30457 SGB63218	0	0.03071
GGB30458 SGB41562	0	0.00241
GGB30461 SGB43530	0	0.09318
GGB30461 SGB43533	0	0.29894
GGB30461 SGB63209	0	0.00315
GGB30463 SGB102197	0	0.16181
GGB30466 SGB43540	0	0.95528
GGB30466 SGB43544	0	0.00785
GGB30466 SGB63211	0	0.01321
GGB30468 SGB43552	0	0.01362
GGB30473 SGB43557	0	0.17993
GGB30620 SGB43754	0	0.16402
GGB30667 SGB43809	0	0.3167
GGB31064 SGB44328	0	0.50223
GGB31109 SGB44380	0	0.44928
GGB31312 SGB44628	0	0.11499
GGB31439 SGB44769	0	0.52772
GGB31701 SGB45057	0	0.2236
GGB3171 SGB4185	0	0.13278
GGB31823 SGB45199	0	0.30646
GGB31839 SGB45217	0.03345	1.40112
GGB31850 SGB45228	0	0.61679
GGB31851 SGB45230	0	0.21088
GGB31853 SGB45233	0	0.10812
GGB3583 SGB21441	0	2.13401
GGB3661 SGB41629	0	0.13443
GGB3793 SGB41498	0	0.17019
GGB42328 SGB59496	0	0.04002
GGB42343 SGB59511	0	0.13431
GGB42344 SGB59512	0	0.09735
GGB42586 SGB59781	0	0.02015
GGB42949 SGB43520	0	0.03062
GGB45293 SGB41002	0	0.39775

GGB45514 SGB63186	0	0.01821
GGB45536 SGB63216	0	0.0775
GGB45650 SGB63364	0	0.0103
GGB45977 SGB41021	0	0.01222
GGB47127 SGB65053	0	0.0713
GGB47180 SGB59839	0	0.02132
GGB47462 SGB65425	0	0.02576
GGB49462 SGB63227	0	0.04215
GGB5207 SGB43496	0	0.04238
GGB54153 SGB41631	0	0.46928
GGB70394 SGB94816	0	0.07439
GGB74395 SGB43523	0	0.06343
GGB74408 SGB43542	0	0.07777
GGB75036 SGB102224	0	0.01305
GGB75053 SGB43494	0	0.11501
GGB75055 SGB43513	0	0.01265
GGB75064 SGB102200	0	0.04921
GGB75064 SGB43546	0	0.0259
GGB75085 SGB102286	0	0.00501
GGB75106 SGB102240	0	0.00683
GGB75900 SGB102322	0	0.03186
GGB75975 SGB102453	0	0.14421
GGB79592 SGB45219	0	0.14268
GGB79731 SGB41725	0	0.30973
GGB79848 SGB41595	0	0.05902
GGB79857 SGB42426	0	1.48303
GGB79872 SGB36958	0	0.33777
GGB79926 SGB43248	0	0.21505
GGB79948 SGB41679	0	0.02542
GGB79989 SGB41645	0	0.00182
GGB80042 SGB43554	0	0.86513
GGB80101 SGB21440	4.41276	1.56761
GGB3583 SGB21441	5.6962	0
Hungateiclostridiaceae bacterium KB18	0	0.03345
Lachnospiraceae bacterium	0	0.03026
Lachnospiraceae bacterium MD308	0	0.02835
Lachnospiraceae unclassified SGB41402	0	0.01033
Lachnospiraceae unclassified SGB41424	0	0.02219
Lactobacillus SGB5158	0	0.02068
Lactobacillus taiwanensis	0.53578	0.01862
Lactococcus SGB40208	0	1.31695

Leuconostoc SGB43006	0	0.07949
Ligilactobacillus_murinus	0.14169	0
Limosilactobacillus SGB43493	0	0.05189
Limosilactobacillus_reuteri	0.14098	0
Methanomethylovoran SGB40959157	0	0.22484
Muribaculaceae bacterium	3.66231	2.52312
Muribaculaceae bacterium Isolate_013_NCI	5.19565	1.82399
Muribaculaceae bacterium_Isolate_110_HZI	0.58142	0.22733
Muribaculum intestinale	2.54524	0.95207
Odoribacter spZ80	0	1.08894
Oscillibacter sp13	0	0.01114
Oscillibacter spCU971	0	0.36961
Oscillospiraceae bacterium	0	0.02386
Parabacteroides goldsteinii	0	0.09265
Paramuribaculum intestinale	0.86322	0.34668
Prevotella spPCJ2	0.13709	5.08024
Rikenellaceae bacterium	0	2.26775
Streptococcus SGB45231	0	0.03049
Turicimona muris	5.07825	1.03866
Zoogloeaceae SGB41465	0	0.00438
**Total**	**100**	**100**

Table S6. Bacterial community structure in ileum and colon samples from SPF mice. Values displayed are relative abundance in "%" at the family level.

SPF bacterial families	Ileum	Colon
Aerococcaceae	0.051	2.23
Akkermansiaceae	0.16	0.13
Atopobiaceae	0.02	0.01
Bacteroidaceae	0.05	4.16
Bacteroidetes phylum families	23.18	14.40
Barnesiellaceae	3.29	0
Brevibacteriaceae	0	0.03
Candidatus_Melainabacteria phylum classified families	0	0.45
Candidatus_Saccharibacteria phylum classified families	0	0.09
Christensenellaceae	0	0.17
Clostridiaceae	0.69	0.77
Desulfovibrionaceae	0.35	11.01
Eggerthellaceae	0	0
Eubacteriales_Family_XIII_Incertae_Sedis	0	0.01
Firmicutes phylum classified families	5.71	21
Lachnospiraceae	1.40	1.97
Lactobacillaceae	0.82	0.20
Muribaculaceae	42.63	15.40
Odoribacteraceae	0	1.09
Peptoniphilaceae	0	0.03
Oscillospiraceae	0	6.92
Prevotellaceae	0.14	5.08

Proteobacteria	0	0.32
Rikenellaceae	0.03	6.62
Ruminococcaceae	0	0.01
Selenomonadaceae	13.54	4.57
Staphylococcaceae	2.60	0.89
Streptococcaceae	0	1.35
Sutterellaceae	5.34	1
Tannerellaceae	0	0.09
Zoogloeaceae	0	0
Sum	**100**	**100**

Table S7: Percentage of non-mouse reads assigned as prophage and bacteriophage designated fractions.

	**Prophages**	**Bacteriophages**
**SPF**	0.63	4.24
**Oligo-MM12**	0.45	2.84
**ASF**	0.80	0.93

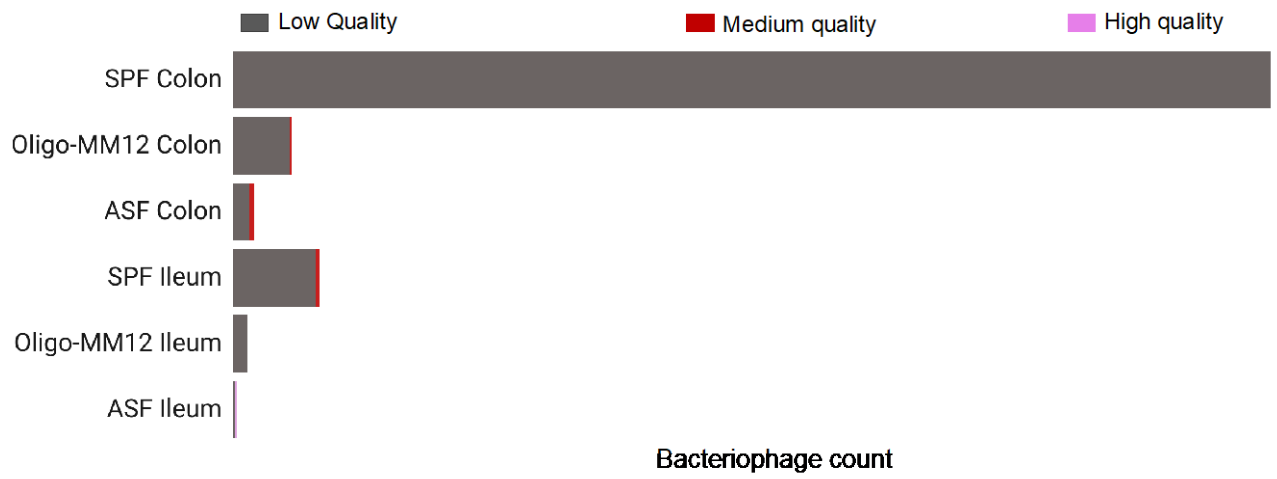

Figure S3: Bacteriophage-designated contigs count in Gnotobiotic Oligo-MM12 and ASF compared to wildtype SPF. Bacteriophage contigs quality was classified based on genome completeness. Red and grey colour bars denote medium and low quality, while pink denotes high quality.

Table S8: List of identified prophages (integrated virus) in SPF and gnotobiotic Oligo-MM12 and ASF mice using PHASTER web tool pipeline (https://phaster.ca/) with a default minimum contig length >2000 bp. Complete prophages designated as 100% intact are highlighted in red, > 70% are questionable prophages denoted in black, while prophages with a completeness score of < 70% denoted as green are considered incomplete prophages.

	**Viral genome hallmark genes**	**Length (kb)**	**Completeness score**	**NCBI Phage name**	**GC%**
**SPF Colon**	terminase, tail	50.1	Questionable (70)	*Lagaffevirus lagaffe*	53.95
	lysin	14.8	Incomplete (20)	*Unahavirus uv1H*	50.51
	tail	8.7	Incomplete (20)	*Caudoviricetes virus*	36.69
	Tail, Integrase	7.7	Incomplete (40)	*Lullwatervirus*	51.42
	Tail, head, portal, terminase	20.3	Questionable (80)	*Vedamuthuvirus BM13*	45.14
	tail,plate,capsid,portal,terminase,integrase	20.1	Complete (100)	*Lilyvirus*	38.97
	Coat, head, tail	13.7	Questionable (70)	*Yongloolinvirus MMP01*	42.86
	Tail, capsid	53	Complete (100)	*Brigitvirus*	45.05
	NA	5.1	Incomplete (10)	*Harrisonvirus*	38.69
	Tail, capsid, portal, protease, terminase	16.2	Complete (100)	Trabyvirinae	48.53
	Portal,tail	15.4	Incomplete (50)	*Secretariatvirus*	51.40
	plate	15.1	Incomplete (20)	*Bixzunavirus*	49.08

	Lysin, tail, terminase	11.1	Incomplete (50)	*Vashvirus lilbooboo*	62.41
	Capsid, terminase,head	22	Incomplete (40)	*Pippivirus lotta*	55.19
	Tail, head, recombinase, terminase	12.5	Incomplete (60)	*Caudoviricetes virus*	47.85
	Capsid, portal, terminase	11.2	Incomplete (40)	*Leicestervirus CD382*	45.87
	NA	11.4	Incomplete (10)	*Arawnvirus arawn*	29.31
	tail	5	Incomplete (20)	*Rimavirus rima.*	43.47
	Capsid, portal, terminase	14.7	Incomplete (40)	*Sherbrookevirus CDHM14*	49.01
	Tail	8.9	Incomplete (20)	*Oengusvirus oengus*	49.34
	Terminase, head	16.3	Incomplete (30)	*Pippivirus pippi*	56.22
	terminase,portal,protease,head,tail	16	Questionable (70)	*Cequinquevirus Ld17*	41.21
	portal,terminase,tail	15.4	Incomplete (60)	*Caudoviricetes virus*	45.22
	Lysin	15.5	Incomplete (20)	*Mushuvirus mushu*	63.07
	head,tail,portal,capsid,plate	26.4	Complete (100)	*Kamchatkavirus AP45*	57.77
**Oligo-MM12 Colon**	tail	9.6	Incomplete (30)	*Caudoviricetes virus*	48.8
	Lysin	9.3	Incomplete (20)	*Mushuvirus mushu*	57.51

**ASF Colon**	NA	17.6	Incomplete (10)	*Caudoviricetes virus*	31.29
	NA	16.3	Incomplete (10)	*Caudoviricetes virus*	31.62
	head,plate	12.4	Incomplete (30)	*Colneyvirus CD505*	23.04
	capsid,tail,head,portal,terminase,recombinase	25.6	Complete (100)	*Sherbrookevirus CD506*	29.62
	head,portal,terminase,tail	12.5	Incomplete (60)	*Caudoviricetes virus*	39.49
**SPF Ileum**	lysin	14.8	Incomplete (20)	*Labanvirus*	50.52
	Tail	21.7	Incomplete (20)	*Muminvirus filifjonk*	42.17
	Recombinase	16	Incomplete (20)	*Baltivirus*	50.14
	head,terminase, capsid	22	Incomplete (40)	*Pippivirus pippi*	55.19
**Oligo-MM12 Ileum**	Integrase, tail	36.4	Incomplete (50)	*Caudoviricetes virus*	40.37
	capsid	11.7	Incomplete (20)	*Caudoviricetes virus*	52.54
**ASF Ileum**	tail,terminase,portal,head,capsid,lysin	36.2	Complete (100)	*Caudoviricetes LfeSau*	38.98
	lysin,tail,capsid	18.8	Incomplete (40)	*Caudoviricetes virus*	41.37

	tail,integrase	5.7	Incomplete (30)	*Caudoviricetes virus*	37.11
	terminase,capsid	10.2	Incomplete (30)	*Caudoviricetes virus*	40.73
	integrase,terminase, portal, head	24.7	Questionable (70)	*Caudoviricetes virus*	39.54

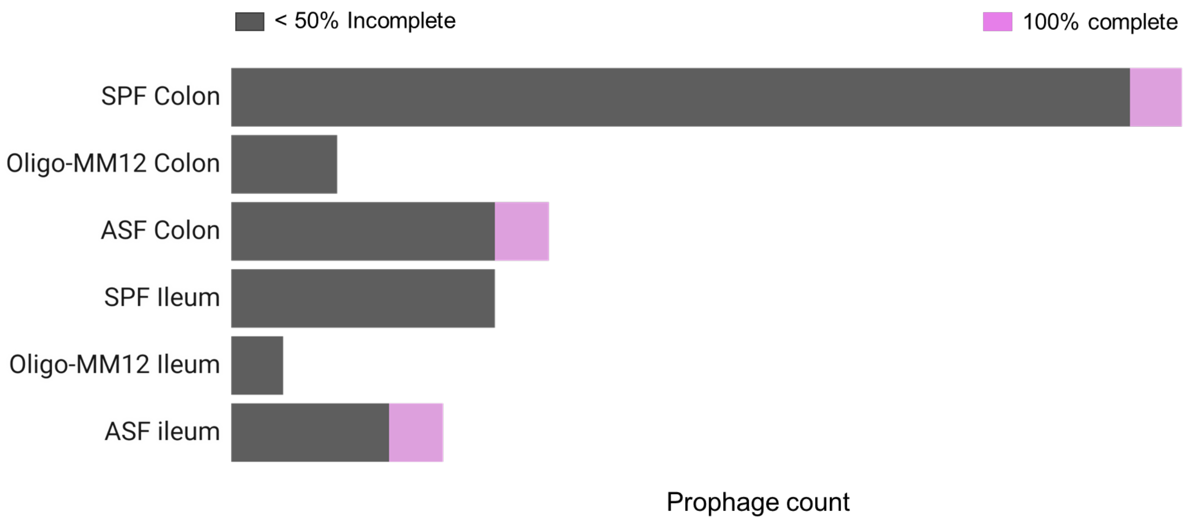

Figure S4: Distribution of prophage contigs according to count and completeness in the colon and ileum of SPF, gnotobiotic Oligo-MM12 and ASF. The identified prophages were classified as complete (100%) and incomplete (< 50%) Grey represents incomplete prophages while pink denotes complete prophages.

Table S9: List of identified prophages (integrated virus) in gnotobiotic Oligo-MM12 and ASF microflora available individual strain sequences (downloaded from NCBI), using PHASTER web tool pipeline (https://phaster.ca/) with a default minimum contig length >2000 bp. Complete prophages designated as 100% intact are highlighted in red, > 70% are questionable prophages denoted in black, while prophages with a completeness score of < 70% denoted as green are considered incomplete prophages (1,2).

**Oligo-MM12**						
**Bacteria sequences from NCBI**	**Family Classification**	**Viral genome key words**	**size(kb)**	**completeness score**	**NCBI phage name**	**GC%**
		Integrase, tail, terminase, capsid	40.6	questionable (90)	Caudoviricetes phage	41.24
		tail, lysin, transposase	50.2	incomplete(60)	Caudoviricetes phage	24.78
		Integrase, head, terminase, capsid	79.5	questionable (80)	Caudoviricetes phage	
Clostridium innocuum	*Lachnospraceae*					
		tail, capsid	14.6	incomplete(40)	Caudoviricetes phage	38.73
		Integrase	29.9	incomplete(20)	Punavirus RCS47	34.91
Enterococcus faecalis	*Enterococcaceae*					
		transposase,head	32.4	incomplete(60)	Gamaleyavirus Bp4	44.87
		Integrase,transposase	31.4	intact(100)	Peduovirus	46.12
Muribaculum intestinale	*Muribaculaceae*					
Akkermansia muciniphila		Integrase	34.9	incomplete(30)	Hollowayvirus F116	55.08
	Akkermansiaceae	none	10.4	incomplete(10)	Caudoviricetes phage	51.82
		tail, transposase, recombinase, portal, lysis	44.5	intact(140)	Caudoviricetes phage	45.55
		Integrase, terminase, capsid, coat	46	questionable (90)	Leicestervirus CD111.	42.34
		tail, fiber	11.4	incomplete(30)	Caudoviricetes phage	42.5
Blautia coccoides	*Lachnospraceae*					
		Integrase, tail, recombinase, transposase	38	questionable (80)	Donellivirus	54.1
		Integrase,transposase,plate	18.6	incomplete(60)	Friunavirus	49.44
		coat, lysis	14.9	incomplete(30)	Caudoviricetes phage	59.19
Acutalibacter muris	*Oscillospiraceae*	tail, transposase	21.1	questionable (90)	Leicestervirus CD111.	57.6

Bacteroides caecimuris	*Bacteroidaceae*	integrase,tail	9.9	incomplete(60)	Caudoviricetes phage	46.78
		tail, transposase	9.5	incomplete(30)	Leicestervirus	42.14
Lactobacillus reuteri	*Lactobacillaceae*	integrase,transposase	34.8	questionable (70)	Lafunavirus	36.41
Flavonifractor plautii	*Oscillospiraceae*	recombinase,head	24.6	incomplete(30)	Caudoviricetes phage	50.12
		portal,capsid	16.3	incomplete(30)	Caudoviricetes phage	51.05
		integrase	16.5	incomplete(20)	Caudoviricetes phage	49.28
		tail,plate,terminase,portal,head	34.2	intact(100)	Caudoviricetes phage	60.17
Bifidobacterium animalis subsp. animalis	*Bifidobacteriaceae*	None				
Turicimonas muris	*Sutterellaceae*	unknown	14.5	incomplete(10)	*Caudoviricetes phage*	*40.5*
		tail,integrase	39.1	incomplete(50)	*Caudoviricetes phage*	*40.57*
		head,terminase	8.8	incomplete(30)	*Caudoviricetes phage*	*44.69*
Clostridium clostridioforme	*Lachnospiraceae*	integrase	26.7	incomplete(30)	*Caudoviricetes phage*	*43.42*
		integrase, tail	26.9	incomplete(30)	*Caudoviricetes phage*	*46.25*
		lysin,tail,plate,capsid,protease,portal,terminase	49.9	intact(150)	*caudoviricetes phage; Toutatisvirus*	*48.09*
		transposase	12.8	incomplete(50)	*Caudoviricetes phage*	*45.6*
		transposase,integrase	29.4	incomplete(40)	*Caudoviricetes; Magadivirus*	*47.34%*
		integrase,transposase,tail	8.7	incomplete(50)	*Caudoviricetes; Peduovirus*	*43.28%*
		recombinase,transposase	31	questionable(70)	*Caudoviricetes Oenococcus phage*	*46.03%*
		transposase,tail	12.9	incomplete(60)	*Caudoviricetes phage*	*44.36%*
		recombinase,transposase	24.3	incomplete(40)	*Caudoviricetes phage*	*48.36%*
		lysin,transposase,integrase	37.2	questionable(70)	*Caudoviricetes phage*	*42.76%*
		tail,head,capsid,portal,terminase,lysin,integrase	42.7	intact(150)	*Caudoviricetes phage*	*47.63%*
		lysin	44.2	questionable(77)	*Caudoviricetes; Mushuvirus*	*51.91%*
		transposase,tail	17.5	incomplete(40)	*unclassified*	*44.27%*
		transposase,tail,integrase	38.3	questionable(80)	*Caudoviricetes phage*	*43.60%*

		none	33.6	incomplete(20)	*Caudoviricetes; Autographiviridae*	*45.02%*
		none	15.5	incomplete(10)	*Caudoviricetes; Kyanoviridae*	*48.10%*
		none	25.3	incomplete(10)	*Caudoviricetes; Guelinviridae*	*50.74%*
		tail	9.9	incomplete(20)	*Caudoviricetes phage*	*45.83*
		transposase	7	incomplete(40)	*Caudoviricetes; Yongloolinvirus*	*42.78*
		integrase	15.7	incomplete(20)	*Caudoviricetes phage*	*50.28*
		integrase,transposase	43.7	intact(100)	*Caudoviricetes phage*	*49.21*
		none	8	incomplete(10)	*Caudoviricetes phage*	*48.33*
		tail,capsid,head,portal	44.2	intact(110)	*Caudoviricetes; Yongloolinvirus*	*49.26*
		lysin	45.6	incomplete(30)	*Caudoviricetes phage*	*49.19*

ASF						
**Bacteria sequences from NCBI**	**Family taxa**	**Viral genome key words**	**Size (kb)**	**completeness score**	**NCBI phage name**	**GC%**
Clostridium spp ASF	*Clostridiaceae*	tail	20.6	incomplete(30)	*Abouovirus*	28.33
		tail, terminase, capsid, portal, head, plate	22.9	intact(110)	*Yongloolinvirus MMP03*	30.14
		Integrase	30.2	incomplete(40)	*Caudoviricetes phage*	31.18
		tail	17.1	incomplete(50)	*Caudoviricetes phage*	32.16
		integrase,recombinase,terminase,portal,head,tail,capsid	41.1	intact(150)	*Sherbrookevirus*	29.55
		integrase,tail	9.2	incomplete(30)	*Phietavirus*	30.34
		tail, capsid	32.1	questionable(70)	*Yongloolinvirus*	35.38
		none	6.1	incomplete(10)	*Saundersvirus*	27.72
		integrase	19.6	incomplete(40)	*Colneyvirus CD505*	29.09
		integrase,head,tail,plate	46.5	intact(100)	*Brigitvirus*	36.26
		none	12.9	incomplete(10)	*Caudoviricetes phage*	30.75
		portal,recombinase,integrase,tail	26.9	questionable(70)	*Caudoviricetes phage*	35.14
		portal,recombinase,tail,integrase	16.2	incomplete(50)	Colneyvirus CD505	29.71
Lactobacillus murinus ASF	*Lactobacillaceae*	transposase,terminase,portal,tail,capsid,plate,lysin	52	intact(150)	*Anamdongvirus*	39.89
		Integrase,transposase,tail	17.3	incomplete(60)	*Caudoviricetes phage*	38.00
		tail, transposase	16	incomplete(40)	*Fernvirus*	39.94
		capsid, transposase	6.8	questionable(70)	*Fernvirus*	37.22
Parabacteriodetes goldsteinii	*Tannerellaceae*	integrase	8.7	incomplete(40)	*Oshimavirus*	42.82
		head,portal,terminase,tail	12.5	incomplete(60)	*Caudoviricetes phage*	39.49

Table S10: Similarity percentage (%) **of** Oligo-MM12 and ASF prophages (integrated virus) to genomes of single bacterial strains used for Oligo-MM12 and ASF to predict their host bacteria using blastn.

	**Prophage-designated contig name**	**Bacteria strains of Gnotobiotic Oligo-MM12 and ASF**	**Family Taxonomic level**	**Similarity (%)**
**Oligo-MM12 Colon**	k141_881,flag=1,multi=6.0000,len=10493:671-10493	*Clostridium innocuum*	*Lachnospiraceae*	100
	k141_2564,flag=1,multi=29.0000,len=11644:381-11419	*Clostridium clostridioforme*	*Lachnospiraceae*	100
**Oligo-MM12 Ileum**	k141_28455,flag=1,multi=49.9999,len=31210:17020-28738	*Akkermansia muciniphila*	*Akkermansiaceae*	92.20
	k141_24561,flag=1,multi=15.0020,len=55382:14360-50825	*Unclassified*		
**ASF Colon**	k141_2793,flag=1,multi=61.4261,len=20498:1547-19217	*Clostridium sp.*	*Clostridiaceae*	83.81
	k141_4731,flag=1,multi=34.0000,len=42346:6795-23099	*Clostridium sp.*	*Clostridiaceae*	84.04
	k141_4731,flag=1,multi=34.0000,len=42346:29024-41515	*Clostridium sp.*	*Clostridiaceae*	84.04
	k141_6678,flag=1,multi=35.0119,len=28816:1397-27046	*Clostridium sp.*	*Clostridiaceae*	81.91
	k141_7003,flag=0,multi=416.0951,len=67939:2322-14050	Unclassified		
**ASF Ileum**	k141_2644,flag=1,multi=28.0000,len=44442:4689-40895	*Lactobacillus murinus*	*Lactobacillaceae*	81.90
	k141_4358,flag=1,multi=27.0000,len=19627:672-19491	*Lactobacillus murinus*	*Lactobacillaceae*	80
	k141_4631, flag=1,multi=15.0000,len=15958:8963-14675	*Lactobacillus murinus*	*Lactobacillaceae*	100
	k141_5131, flag=1,multi=26.0720,len=16007:150-10380	*Lactobacillus murinus*	*Lactobacillaceae*	100
	k141_6867,flag=1,multi=27.0000,len=24904:167-24904	*Lactobacillus murinus*	*Lactobacillaceae*	81.34

a)

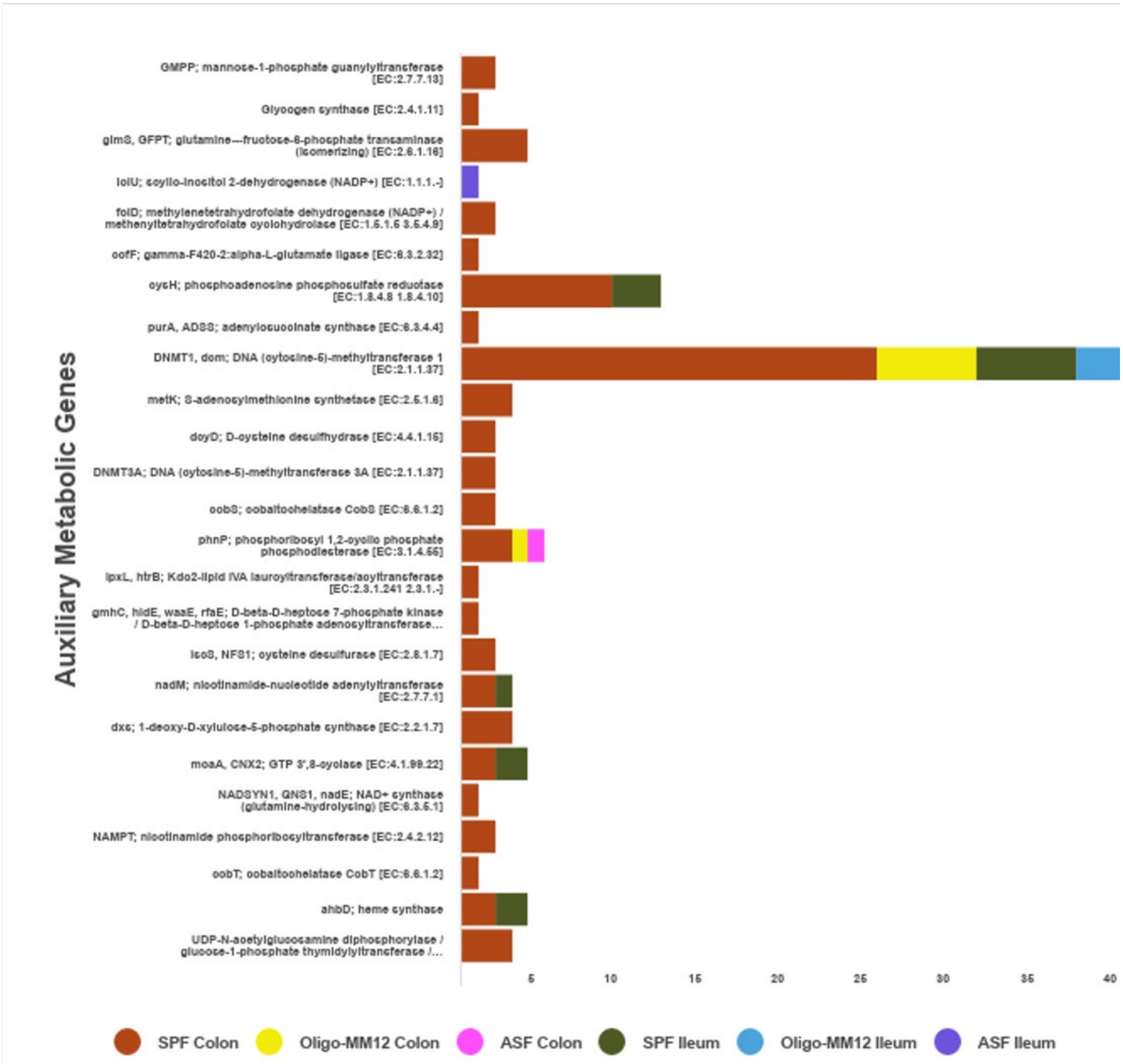

Figure S5: Bacteriophages associated auxiliary metabolic genes copy count in Gnotobiotic Oligo-MM12 and ASF compared to SPF.
